# What's Race Got to Do With It? CRP Levels in Immune Mediated Skin Diseases: Considerations for Hidradenitis Suppurativa

**DOI:** 10.3389/fimmu.2022.847050

**Published:** 2022-03-29

**Authors:** Chidubem A. V. Okeke, Jonathan P. Williams, Callyn U. Iwuala, Pearl K. Igwe, Raveena Khanna, Jessica D. Perry, Ginette A. Okoye, Angel S. Byrd

**Affiliations:** ^1^ Howard University College of Medicine, Washington, DC, United States; ^2^ Cooper Medical School of Rowan University, Camden, NJ, United States; ^3^ Department of Psychiatry, Creighton University School of Medicine, Phoenix, AZ, United States; ^4^ Department of Dermatology, Howard University College of Medicine, Washington, DC, United States

**Keywords:** hidradenitis suppurativa, psychosocial impact, skin of color, inflammatory skin disease, immune mediated skin disease, dermatology, diversity, health care disparities

## Abstract

Currently, there is a lack of racial/ethnic heterogeneity in research databases, exposing a systematic issue in studies exploring inflammation-mediated diseases, such as hidradenitis suppurativa (HS). HS is a chronic inflammatory skin condition that disrupts normal structure and functioning of terminal hair follicles, resulting in the formation of recurrent abscesses, nodules, and sinus tracts within intertriginous regions. Studies have described higher serum levels of inflammation-mediated C-reactive protein (CRP) in patients with HS, a disease that predominantly affects skin of color (SOC) populations. Herein, we explore the role of CRP levels in the context of HS disease presentation, management, and psychosocial implications in SOC patients to determine existing disparities in research studies.

## Introduction

Skin of color (SOC) refers to individuals of African, Asian, Native American, Middle Eastern, and Hispanic backgrounds (1). According to the 2020 United States (US) Census, these persons collectively constitute nearly half of the population (2). Among the US and United Kingdom (UK) populations, the Black community accounts for 13.4% of 328 million and 3.3% of 56.1 million, respectively (3). Despite being one of the largest minority groups in both countries, this population is marginally included in research.

A recent article by Nagar et al. revealed higher serum levels of inflammation-mediated C-reactive protein (CRP) in UK populations composed largely of older, Black females. Notably, the sample included only 6,456 (0.1%) Black subjects and 426,842 (98.5%) White subjects (4). Compared to census demographics reported during the same time period (2006-2011), this sampled population underrepresents Black communities (3). Their findings suggest that socio-environmental factors play a consequential role in explaining these disparities. Yet, the lack of racial/ethnic heterogeneity in their dataset demonstrates a systematic issue in studies exploring inflammation-mediated diseases. This fundamental representation gap limits our understanding of disease biomarker influence on inflammatory skin conditions that disproportionately affect minority patients.

CRP is an acute phase reactant produced by the liver during inflammation. With pro-inflammatory and anti-inflammatory properties, CRP is essential in the clearance of foreign antigens and damaged cells (5). Traditionally, it has been used as a biomarker for infectious and cardiovascular events (6), but various studies also demonstrate a correlation between CRP levels and disease severity in inflammatory skin conditions (7–9) such as HS (10). Research shows that socioeconomic and psychosocial factors likely contribute to variations in CRP levels between Blacks and Whites. Low-income status and smoking activity contributed most to elevated CRP levels among older Black males, whereas obesity mainly contributed to elevated CRP levels in older Black females (11). Moreover, self-reported daily and lifetime discrimination by Blacks/African Americans (AA) correlated with increased CRP levels (12).

Here, we highlight the limited correlative research on CRP in SOC HS patients to address this population’s existing health disparities and poor psychosocial outcomes. The present study found that though differences in CRP levels have been related to HS severity, there is paucity of research exploring its correlation to race in the disease. Our aim is to encourage dialogue about the underrepresentation of Blacks/AA within CRP as well as HS investigations, underscoring the need for diversity in clinical and biomedical studies to produce generalizable data and results.

## Hidradenitis Suppurativa

HS is a chronic inflammatory skin condition that disrupts normal structure and functioning of terminal hair follicles. It is characterized by the formation of recurrent abscesses, nodules, and sinus tracts in intertriginous regions, with severe complications including soft tissue infection, lymphedema, and sepsis (13–15). Although the pathogenesis is not entirely understood, studies show that HS is three times more prevalent in Blacks/AA compared to Whites (16, 17). Yet, there is a scarcity of diverse representation in research examining CRP's role in inflammatory diseases despite its strong association and increased levels in SOC patients.

Quantitative evaluation has revealed higher CRP levels among HS patients than matched controls, independent of BMI and smoking status (10, 18). Moreover, CRP levels were found to be an independent predictor of Hurly Stage III, the most severe form of the disease (19). In patients with moderate-to-severe HS, Adalimumab treatment led to significant improvement in the clinical inflammatory load. These patients also showed reductions in serum CRP levels from baseline following treatment (20). All of these studies account for various predisposing factors, such as smoking status, obesity (BMI), gender, and age while neglecting to consider race (10, 19, 21). **Table 1** shows a summary of the information extracted from these studies. Though several studies suggest CRP as a potential biomarker for HS, the scarcity of representation of SOC in such studies prevents the generalizability of this data. Monitoring race in studies such as these can further explain extenuating aspects that cause minority patients to be affected by common disease processes differently.

**Table 1:** Summary of studies exploring HS and CRP.

Study	Year	Race Explored (Y/N)	Total Patients (n)	Black (%)	White (%)	Male (n)	Female (n)	Mean Age (Years)	Type of Study	Main finding about CRP
Study 1 (22)	2020	N	88 (44 HS patients, 44 age and gender matched controls)	*	*	28	14	31.5 HS, 33.5 control	Case-control study	Serum C-reactive protein (CRP) levels are higher in Hidradenitis Suppurativa (HS) patients than in controls; significantly higher levels of serum CRP were even found in the patients with severe disease manifestations. The study showed CRP levels to be associated with the severity and stage of HS, therefore serum CRP level is a valuable parameter in patient evaluation and management.
Study 2 (23)	2019	N	25 total HS patients	*	*	11	14	41	Retrospective chart review	A median CRP level of 11.5 mg/dl (~1.5x upper limit of normal) in HS patients found across all Hurley stages, with increased levels found with higher Hurley staging. CRP of Female patients with Hurley Stage II vs. III was 14.4 mg/dl and 23 mg/dl respectively. For men with Hurley Stage II vs. III, CRP levels were 6.45 mg/dl and 13.57 respectively.
Study 3 (24)	2017	N	96 (74 HS, 22 control)	*	*	36	38	37.4	Case-control study	Serum proinflammatory cytokines, CRP, and ESR are increased in relation to the clinical inflammatory activity of patients with HS compared with healthy controls. Serum IL-6, CRP, and ESR are effective biomarkers for evaluating HS severity.
Study 4 (25)	2018	N	80 (40 HS patients, 40 aged and gender-matched controls)	*	*	23	17	35.4	Case-control study	There was a marked increase in the serum high sensitivity CRP (hs-CRP) level—independent of BMI and smoking status—in the HS patients, as compared to the controls.
Study 5 (26)	2020	N	140 (40 HS patients, 100 age and gender-matched controls)	*	*	HS patients: 21 Healthy controls: 53	HS patients: 19. Healthy controls: 47	41.7 HS, 42.2 control	Cross sectional, case-control study	hs-CRP and epicardial fat thickness (EFT) were significantly higher in HS patients compared to controls. There were positive correlations between EFT and the duration of the disease, hs-CRP, and Hurley stage. hs-CRP, body mass index, and EFT≥5.9 mm were independent predictors of severe disease.
Study 6 (27)	2017	N	13 total HS patients	*	*	4	9	43 non-responders, 33 responders	Prospective pilot study	This study aimed to determine if the baseline inflammatory profile of patients with HS could predict the response to treatment with Infliximab (IFX). High baseline levels of IL-6 and hs-CRP were predictive of nonresponse to IFX. Initial levels of hs-CRPand IL-6 are potential response markers for IFX treatment in HS.
Study 7 (20)	2018	N	38 total (19 HS patients, 19 healthy controls)	*	*	HS patients: 11. Healthy Controls: 9	HS patients: 8. Healthy controls: 10	45.6 HS, 35.7 control	Prospective, single-centre case control study	Prior to treatment with Adalimumab, HS patients showed significantly increased levels of iL-6, IL-8, IL-10, ESR, soluble TNF receptor II (sTNF-RII), and C-reactive protein (CRP). After treatment, the circulating levels of all of the listed inflammatory markers decreased significantly. The decrease in IL-6, IL-8, ESR, sTNF-RII, and CRP were significantly correlated with clinical improvements according to the modified HS score (mHSS).
Study 8 (10)	2015	N	50 HS patients, 250 age and gender matched controls	*	*	HS patients: 12. Healthy Controls: 9	HS patients: 38. Healthy controls: 47	42.9 HS, 42.2 control	Retrospective chart review	HS patients were found to have a higher inflammatory load compared to other dermatological patients as reflected by increased levels of lymphocytes, neutrophilocytes and C-reactive protein. Increased Hurley stage was positively correlated to increased CRP category, increased neutrophils, and increased N/L ratio.
Study 9 (28)	2015	N	104 total HS patients	*	*	26	78	37.7	Retrospective chart review	Median CRP level was significantly different among the 3 Hurley groups and increased with the degree of severity. There were significant positive correlations between CRP levels and neutrophil count with modified Hidradenitis Suppurativa Score. CRP was a significant independent predictor for Hurley Stage III. CRP and body mass index were significant independent predictors for severe disease according to modified Hidradenitis Suppurativa Score.
Study 10 (29)	2021	N	50 (26 HS patients, 24 healthy controls)	*	*	HS patients: 18. Healthy Controls: 17	HS patients: 8. Healthy controls: 7	35.12	Prospective, case control study	HS patients had significantly higher hs-CRP levels than controls which decreased following treatment.
Study 11 (30)	2016	N	124 total (62 HS patients, 62 matched controls)	*	*	HS patients: 27. Healthy Controls: 22	HS patients: 35 Healthy controls: 40	40.6 HS, 46.6 control	Prospective observation and analytical study	Elevated neutrophil-to-lymphocyte ratio (NLR), Hs-CRP and erythrocyte sedimentation rate were more frequent in patients with HS.
Study 12 (31)	2010	N	8 total HS patients	*	*	3	5	42	Prospective case control	Eight patients with severe hidradenitis were treated for 1 year with adalimumab in a standard regimen and were subsequently followed for 1 year. All patients improved within 4-6 weeks and laboratory parameters of C-reactive protein (CRP) and leukocyte count reduced significantly during treatment. The mean CRP value reduced from 42.5 to 12.8 mg/l at 6 weeks and to 5.2 at 6 months.
Study 13 (32)	2021	N	166 HS patients, 124 healthy controls	*	*	HS patients: 98. Healthy Controls: 62	HS patients: 68 Healthy controls: 62	35.8. HS, 34.0 control	Retrospective study	MCV (Mean corpuscular volume), RDW (Red cell distribution width), and CRP showed a significant positive correlation with disease severity. This study shows that CRP remains significant in evaluating HS disease activity compared to alternative inflammation biomarkers.
Study 14 (33)	2016	Y	32 hospital cases of HS individuals (HS-HOSP group), 430 HS individuals found in the general population (HS-POP group), and 20,780 controls	unknown	97% of participants were caucasian	HS-hosp patients: 22, HS-POP patients: 32, Controls: 46	HS-hosp patients: 78, HS-POP patients: 68. Controls 54	HS-hosp patients 40.6, HS-POP patients: Controls 46.6	Comparative cross-sectional study	The study aimed to investigate the status of inflammation and leukocyte profile in the peripheral blood of HS patients. They investigated blood samples of high-sensitivity C-reactive protein (hs-CRP) and leukocyte profile in hospital-treated HS patients (HS-HOSP), self-reported population-based HS patients (HS-POP) and population controls. An age-sex-adjusted analysis revealed a significantly higher hs-CRP for both HS groups compared to controls
Study 15 (34)	2016	N	43 total HS patients (22 treated with antibiotics and HBOT, 21 antibiotics only)	*	*	18	25	34.0 antibiotics and HBOT, 37.5 antibiotics only	Prospective, single center, randomized controlled clinical trial	Study aimed to evaluate efficacy of HBOT as an adjunctive therapy in patients with HS receiving systemic rifampicin and clindamycin. Groups were comparable with respect to age, BMI, gender, and smoking habits. ESR and CRP levels were gathered at baseline and at 4 and 10 wks of treatment. Patients in the HBOT group showed a decreased from baseline parameters including CRP (72.7%). Adjunctive HBOT significantly improved antibiotic treatment of HS.

The symbol * represents missing data.

Oversight of this inequality maintains and exacerbates existing psychosocial outcomes of Black HS patients. These communities report a lower health-related quality of life, which is attributed to debilitating chronic pain, poor mental health and diminished self-sufficiency (35–38). Phenotypic manifestations, including disfiguring nodules with malodorous discharge, perpetuate social stigma, low self-esteem and self-isolation. This is implicated in the disproportionate rates of depression, anxiety, substance use, and suicidal ideation among this population (37, 39–43). Interestingly, patients with major depressive disorder exhibit increased peripheral blood concentrations of CRP (44, 45), and elevated CRP levels predict resistance to standard antidepressant therapies (44, 46, 47). Black/AA patients are more likely to have severe HS with elevated CRP levels (48, 49). Thus, neglecting these communities hinders quality of life while deepening existing disparities (49, 50).

## Discussion

Inflammatory skin conditions like HS have distinct features in SOC populations, and CRP is a commonly utilized biomarker that is linked to disease activity. Compared to individuals without disease, CRP levels are significantly elevated in individuals with HS. Despite the vast evidence of more severe disease manifestations among Black populations, there is a gross underrepresentation of racial minorities throughout clinical and biomedical research even in diseases that disproportionally affect SOC patients.

Addressing the racial disparity in clinical and biomedical research studies, databases, and biorepositories is therefore critical to reducing the disease burden experienced by SOC patients. Researchers should accurately report race/ethnicity data and establish race-matched healthy controls. Understanding mediators of inflammatory skin diseases among Black patients may reveal pertinent risk factors and optimal therapeutic strategies. This will inform tailored interventions to appropriately address the aforementioned psychological burdens, as this lack of diversity has culminated in poorer outcomes among vulnerable populations. While limitations associated with inadequate diversity in data collection have been established, little has been done to revolutionize change. Therefore, it is imperative that academic researchers prioritize racial/ethnic diversity during patient recruitment as well as biospecimen collection and analysis.

## Data Availability Statement

The original contributions presented in the study are included in the article material. Further inquiries can be directed to the corresponding author.

## Author Contributions

CO, JW, CI, PI, and AB identified the gap in the field and conceptualized the overarching idea. CO, JW, CI, and PI collected and summarized the data from literature searches and drafted the manuscript. RK, JP, GO, and AB provided critical review and revised this manuscript. All authors contributed to the article and approved the submitted version.

## Funding

The publishing of this work was supported by the Skin of Color Society Career Development Award (ASB).

## Conflict of Interest

The authors declare that the research was conducted in the absence of any commercial or financial relationships that could be construed as a potential conflict of interest.

## Publisher’s Note

All claims expressed in this article are solely those of the authors and do not necessarily represent those of their affiliated organizations, or those of the publisher, the editors and the reviewers. Any product that may be evaluated in this article, or claim that may be made by its manufacturer, is not guaranteed or endorsed by the publisher.
